# 4-(2-Cyano­ethyl­sulfan­yl)-5′-(pyridin-4-yl)tetra­thia­fulvalene

**DOI:** 10.1107/S1600536811018800

**Published:** 2011-05-20

**Authors:** Haiyun Li, Guannan Wang, Xunwen Xiao

**Affiliations:** a Department of Chemical Engineering, Ningbo University of Technology, Ningbo 315016, People’s Republic of China; b Department of Chemistry and Chemical Engineering, Taiyuan University of Technology, Taiyuan, People’s Republic of China

## Abstract

In the title compound, C_14_H_10_N_2_S_5_ [systematic name; 3-({2-[4-(pyridin-4-yl)-2*H*-1,3-dithiol-2-yl­idene]-2*H*-1,3-dithiol-4-yl}sul­fan­yl)propane­nitrile], all of the non-H atoms except for the cyano­ethyl­sulfanyl group, are approximately coplanar [maxium deviation = 0.090 (3) Å]. The two five-membered 1,3-dithiole rings are twisted by 2.6 (2)°. Weak inter­molecular S⋯S inter­actions occur [3.586 (4) and 3.530 (4) Å].

## Related literature

For background to the chemistry of prridine-based tetra­thia­fulvalenes, see: Fabre (2004 ▶); Zhu *et al.* (2007 ▶). For the preparation of the title compound, see: Jia *et al.* (2001 ▶); Zhu *et al.* (2010 ▶). For related structures, see: Han *et al.* (2007 ▶); Zhao *et al.* (2008 ▶).
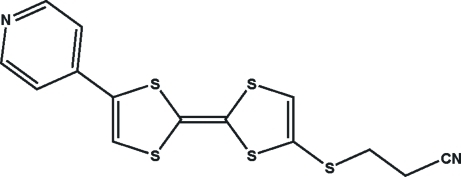


## Experimental

### 

#### Crystal data

C_14_H_10_N_2_S_5_
*M* *_r_* = 366.54
Monoclinic, 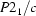

*a* = 14.6231 (18) Å
*b* = 10.7197 (12) Å
*c* = 9.9211 (12) Å
β = 94.775 (4)°
*V* = 1549.8 (3) Å^3^
*Z* = 4
Mo *K*α radiation
μ = 0.74 mm^−1^
*T* = 223 K
0.50 × 0.20 × 0.20 mm

#### Data collection

Rigaku Saturn diffractometer
Absorption correction: multi-scan (*REQAB*; Jacobson, 1998 ▶) *T*_min_ = 0.613, *T*_max_ = 0.856
7658 measured reflections
2869 independent reflections
2268 reflections with *I* > 2σ(*I*)
*R*_int_ = 0.043

#### Refinement

*R*[*F* ^2^ > 2σ(*F* ^2^)] = 0.061
*wR*(*F* ^2^) = 0.126
*S* = 1.11
2869 reflections
191 parameters
H-atom parameters constrained
Δρ_max_ = 0.51 e Å^−3^
Δρ_min_ = −0.32 e Å^−3^

### 

Data collection: *CrystalClear* (Rigaku, 2005) ▶; cell refinement: *CrystalClear*; data reduction: *CrystalStructure* (Rigaku, 2005); program(s) used to solve structure: *SHELXS97* (Sheldrick, 2008 ▶); program(s) used to refine structure: *SHELXL97* (Sheldrick, 2008 ▶); molecular graphics: *ORTEP-3* (Farrugia, 1997 ▶); software used to prepare material for publication: *SHELXTL* (Sheldrick, 2008 ▶).

## Supplementary Material

Crystal structure: contains datablocks I, global. DOI: 10.1107/S1600536811018800/ng5167sup1.cif

Structure factors: contains datablocks I. DOI: 10.1107/S1600536811018800/ng5167Isup2.hkl

Supplementary material file. DOI: 10.1107/S1600536811018800/ng5167Isup3.cml

Additional supplementary materials:  crystallographic information; 3D view; checkCIF report

## Figures and Tables

**Fig. 1.**
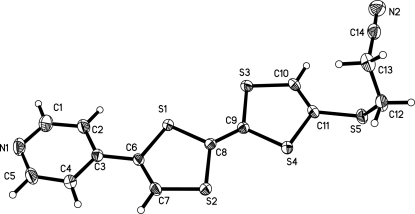
The molecular structure of the title compound.Displacement ellipsoids are drawn at the 30% probability level.

**Fig. 2.**
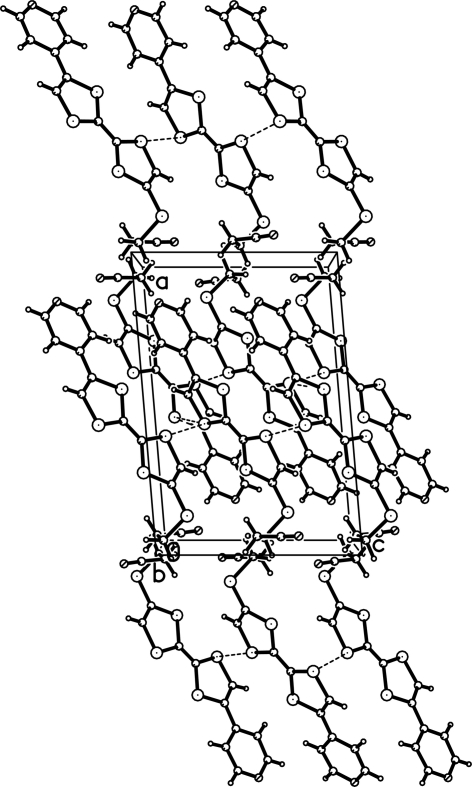
The crystal packing diagram view along the crystallographic b-axis.

## supplementary crystallographic information

### Comment

Tetrathiafulvalene (TTF) and its derivatives are strong electron donors (D). Many electron acceptors (A) have been connected to TTF to afford electron D—A system to build molecular level devices, such as molecular rectifiers and molecular switches. In order to obtain materials in molecular electronics, currently, our research is focus on the synthesis and crystal structures of TTF derivatives. In the title compound, the substituent group of the TTF core are located in opposite direction, resulting in chair-like molecular comformations. All bonds lengths and bond angles are found to within the range for neutral TTF (Han *et al.* 2007). In addition, the pyridyl group and tetrathiafulvalene motif are coplanar, the non-H atoms of the two group lie on a plan [maxium deviation is 0.0908 (33)?Å] (Fig.1). Inter-molecular interaction involving S(2)···S(1) 3.586 (4) Å and S(2)···S(3) 3.530 (4) Å are present consolidating the crystal packing. (Fig.2).

### Experimental

The title compound was prepared according to the literature (Jia *et al.*,2001) (Zhu *et al.*,2007). Red crystals were obtained from slow evaporation of a dichloromethane solution at room temperature.

### Refinement

H atoms were positioned geometrically [C–H = 0.93–0.98 Å] and refined using a riding model, with *U*_iso_(H) = 1.2Ueq(C).

### Crystal data

**Table:**

C_14_H_10_N_2_S_5_	*F*(000) = 752
*M**_r_* = 366.54	*D*_x_ = 1.571 Mg m^−^^3^
Monoclinic, *P*2_1_/*c*	Mo *K*α radiation, λ = 0.71075 Å
Hall symbol: -P 2ybc	Cell parameters from 5787 reflections
*a* = 14.6231 (18) Å	θ = 3.1–27.5°
*b* = 10.7197 (12) Å	µ = 0.74 mm^−^^1^
*c* = 9.9211 (12) Å	*T* = 223 K
β = 94.775 (4)°	Block, red
*V* = 1549.8 (3) Å^3^	0.50 × 0.20 × 0.20 mm
*Z* = 4	

### Data collection

**Table:**

Rigaku Saturn diffractometer	2869 independent reflections
Radiation source: fine-focus sealed tube	2268 reflections with *I* > 2σ(*I*)
graphite	*R*_int_ = 0.043
Detector resolution: 14.63 pixels mm^-1^	θ_max_ = 25.5°, θ_min_ = 3.1°
ω scans	*h* = −17→16
Absorption correction: multi-scan (REQAB; Jacobson, 1998)	*k* = −12→12
*T*_min_ = 0.613, *T*_max_ = 0.856	*l* = −12→9
7658 measured reflections	

### Refinement

**Table:**

Refinement on *F*^2^	Primary atom site location: structure-invariant direct methods
Least-squares matrix: full	Secondary atom site location: difference Fourier map
*R*[*F*^2^ > 2σ(*F*^2^)] = 0.061	Hydrogen site location: inferred from neighbouring sites
*wR*(*F*^2^) = 0.126	H-atom parameters constrained
*S* = 1.11	*w* = 1/[σ^2^(*F*_o_^2^) + (0.0484*P*)^2^ + 1.3388*P*] where *P* = (*F*_o_^2^ + 2*F*_c_^2^)/3
2869 reflections	(Δ/σ)_max_ < 0.001
191 parameters	Δρ_max_ = 0.51 e Å^−^^3^
0 restraints	Δρ_min_ = −0.32 e Å^−^^3^

### Special details

**Table:**

Geometry. All e.s.d.'s (except the e.s.d. in the dihedral angle between two l.s. planes) are estimated using the full covariance matrix. The cell e.s.d.'s are taken into account individually in the estimation of e.s.d.'s in distances, angles and torsion angles; correlations between e.s.d.'s in cell parameters are only used when they are defined by crystal symmetry. An approximate (isotropic) treatment of cell e.s.d.'s is used for estimating e.s.d.'s involving l.s. planes.
Refinement. Refinement of *F*^2^ against ALL reflections. The weighted *R*-factor *wR* and goodness of fit *S* are based on *F*^2^, conventional *R*-factors *R* are based on *F*, with *F* set to zero for negative *F*^2^. The threshold expression of *F*^2^ > σ(*F*^2^) is used only for calculating *R*-factors(gt) *etc*. and is not relevant to the choice of reflections for refinement. *R*-factors based on *F*^2^ are statistically about twice as large as those based on *F*, and *R*- factors based on ALL data will be even larger.

### Fractional atomic coordinates and isotropic or equivalent isotropic displacement parameters (Å^2^)

**Table:**

	*x*	*y*	*z*	*U*_iso_*/*U*_eq_	
S1	0.45140 (7)	0.17432 (9)	0.11234 (11)	0.0324 (3)	
S2	0.57042 (7)	−0.02950 (10)	0.22543 (11)	0.0362 (3)	
S3	0.61033 (7)	0.25532 (10)	−0.08148 (12)	0.0377 (3)	
S4	0.72869 (7)	0.05022 (10)	0.03146 (11)	0.0361 (3)	
S5	0.87437 (8)	0.10167 (11)	−0.16139 (12)	0.0438 (3)	
N1	0.1558 (3)	0.1902 (4)	0.3981 (5)	0.0555 (11)	
N2	0.9396 (3)	0.4512 (4)	−0.1998 (5)	0.0619 (12)	
C1	0.1881 (3)	0.2455 (5)	0.2923 (6)	0.0601 (14)	
H1	0.1532	0.3107	0.2508	0.072*	
C2	0.2690 (3)	0.2145 (4)	0.2386 (5)	0.0459 (12)	
H2	0.2865	0.2562	0.1615	0.055*	
C3	0.3240 (3)	0.1224 (4)	0.2984 (4)	0.0323 (9)	
C4	0.2907 (3)	0.0618 (4)	0.4090 (5)	0.0451 (12)	
H4	0.3242	−0.0035	0.4528	0.054*	
C5	0.2082 (3)	0.0989 (5)	0.4532 (5)	0.0529 (14)	
H5	0.1873	0.0568	0.5278	0.063*	
C6	0.4131 (3)	0.0883 (4)	0.2484 (4)	0.0321 (9)	
C7	0.4680 (3)	−0.0036 (4)	0.2963 (4)	0.0345 (10)	
H7	0.4512	−0.0538	0.3680	0.041*	
C8	0.5581 (2)	0.0973 (3)	0.1123 (4)	0.0284 (9)	
C9	0.6232 (3)	0.1293 (4)	0.0326 (4)	0.0298 (9)	
C10	0.7170 (3)	0.2350 (4)	−0.1449 (4)	0.0348 (10)	
H10	0.7363	0.2886	−0.2120	0.042*	
C11	0.7705 (3)	0.1427 (4)	−0.0967 (4)	0.0350 (10)	
C12	0.9586 (3)	0.1601 (5)	−0.0324 (5)	0.0484 (12)	
H12A	0.9549	0.1103	0.0498	0.058*	
H12B	1.0199	0.1478	−0.0632	0.058*	
C13	0.9480 (3)	0.2965 (5)	0.0034 (5)	0.0511 (13)	
H13A	0.8915	0.3064	0.0491	0.061*	
H13B	0.9995	0.3207	0.0676	0.061*	
C14	0.9445 (3)	0.3823 (5)	−0.1136 (5)	0.0458 (12)	

### Atomic displacement parameters (Å^2^)

**Table:**

	*U*^11^	*U*^22^	*U*^33^	*U*^12^	*U*^13^	*U*^23^
S1	0.0303 (5)	0.0311 (5)	0.0363 (6)	0.0025 (4)	0.0064 (4)	0.0022 (4)
S2	0.0359 (6)	0.0343 (6)	0.0396 (7)	0.0060 (5)	0.0090 (5)	0.0060 (5)
S3	0.0350 (6)	0.0345 (6)	0.0439 (7)	0.0004 (5)	0.0048 (5)	0.0076 (5)
S4	0.0306 (5)	0.0372 (6)	0.0413 (7)	0.0026 (5)	0.0079 (5)	0.0050 (5)
S5	0.0359 (6)	0.0507 (7)	0.0469 (8)	−0.0030 (5)	0.0165 (5)	−0.0037 (6)
N1	0.036 (2)	0.063 (3)	0.070 (3)	−0.005 (2)	0.020 (2)	−0.014 (2)
N2	0.069 (3)	0.056 (3)	0.062 (3)	0.004 (2)	0.014 (2)	0.003 (2)
C1	0.036 (3)	0.066 (3)	0.080 (4)	0.007 (2)	0.011 (3)	0.004 (3)
C2	0.037 (2)	0.051 (3)	0.051 (3)	0.003 (2)	0.010 (2)	0.010 (2)
C3	0.027 (2)	0.032 (2)	0.037 (3)	−0.0060 (17)	0.0039 (18)	−0.0083 (18)
C4	0.044 (3)	0.042 (3)	0.051 (3)	0.000 (2)	0.017 (2)	0.002 (2)
C5	0.047 (3)	0.056 (3)	0.060 (3)	−0.013 (3)	0.025 (3)	−0.007 (3)
C6	0.031 (2)	0.030 (2)	0.036 (2)	−0.0051 (18)	0.0087 (18)	−0.0032 (18)
C7	0.037 (2)	0.038 (2)	0.030 (2)	−0.0037 (19)	0.0103 (18)	0.0014 (19)
C8	0.0252 (19)	0.027 (2)	0.033 (2)	−0.0019 (16)	−0.0001 (17)	−0.0029 (17)
C9	0.030 (2)	0.030 (2)	0.030 (2)	−0.0032 (17)	0.0018 (17)	−0.0028 (17)
C10	0.036 (2)	0.039 (2)	0.030 (2)	−0.0086 (19)	0.0072 (19)	0.0033 (19)
C11	0.031 (2)	0.039 (2)	0.036 (3)	−0.0080 (19)	0.0085 (19)	0.000 (2)
C12	0.031 (2)	0.063 (3)	0.052 (3)	0.000 (2)	0.009 (2)	0.007 (3)
C13	0.041 (3)	0.069 (3)	0.043 (3)	−0.010 (2)	0.007 (2)	−0.007 (3)
C14	0.039 (3)	0.050 (3)	0.049 (3)	−0.003 (2)	0.010 (2)	−0.013 (3)

### Geometric parameters (Å, °)

**Table:**

S1—C6	1.764 (4)	C3—C4	1.398 (6)
S1—C8	1.765 (4)	C3—C6	1.478 (5)
S2—C7	1.729 (4)	C4—C5	1.376 (6)
S2—C8	1.762 (4)	C4—H4	0.9400
S3—C10	1.744 (4)	C5—H5	0.9400
S3—C9	1.762 (4)	C6—C7	1.333 (6)
S4—C9	1.761 (4)	C7—H7	0.9400
S4—C11	1.761 (4)	C8—C9	1.333 (5)
S5—C11	1.753 (4)	C10—C11	1.326 (6)
S5—C12	1.812 (5)	C10—H10	0.9400
N1—C1	1.327 (7)	C12—C13	1.515 (6)
N1—C5	1.331 (7)	C12—H12A	0.9800
N2—C14	1.128 (6)	C12—H12B	0.9800
C1—C2	1.377 (6)	C13—C14	1.479 (7)
C1—H1	0.9400	C13—H13A	0.9800
C2—C3	1.377 (6)	C13—H13B	0.9800
C2—H2	0.9400		
			
C6—S1—C8	95.29 (18)	S2—C7—H7	120.3
C7—S2—C8	95.10 (19)	C9—C8—S2	122.4 (3)
C10—S3—C9	94.88 (19)	C9—C8—S1	123.7 (3)
C9—S4—C11	95.20 (19)	S2—C8—S1	113.8 (2)
C11—S5—C12	102.3 (2)	C8—C9—S4	123.3 (3)
C1—N1—C5	115.0 (4)	C8—C9—S3	122.3 (3)
N1—C1—C2	124.8 (5)	S4—C9—S3	114.3 (2)
N1—C1—H1	117.6	C11—C10—S3	118.8 (3)
C2—C1—H1	117.6	C11—C10—H10	120.6
C3—C2—C1	119.8 (4)	S3—C10—H10	120.6
C3—C2—H2	120.1	C10—C11—S5	123.9 (3)
C1—C2—H2	120.1	C10—C11—S4	116.8 (3)
C2—C3—C4	116.2 (4)	S5—C11—S4	119.1 (2)
C2—C3—C6	122.2 (4)	C13—C12—S5	115.0 (3)
C4—C3—C6	121.6 (4)	C13—C12—H12A	108.5
C5—C4—C3	119.2 (4)	S5—C12—H12A	108.5
C5—C4—H4	120.4	C13—C12—H12B	108.5
C3—C4—H4	120.4	S5—C12—H12B	108.5
N1—C5—C4	124.9 (5)	H12A—C12—H12B	107.5
N1—C5—H5	117.6	C14—C13—C12	114.5 (4)
C4—C5—H5	117.6	C14—C13—H13A	108.6
C7—C6—C3	125.8 (4)	C12—C13—H13A	108.6
C7—C6—S1	116.0 (3)	C14—C13—H13B	108.6
C3—C6—S1	118.2 (3)	C12—C13—H13B	108.6
C6—C7—S2	119.3 (3)	H13A—C13—H13B	107.6
C6—C7—H7	120.3	N2—C14—C13	177.0 (5)
			
C5—N1—C1—C2	0.3 (8)	C6—S1—C8—S2	6.1 (2)
N1—C1—C2—C3	−2.1 (8)	S2—C8—C9—S4	−0.5 (5)
C1—C2—C3—C4	2.7 (7)	S1—C8—C9—S4	−178.9 (2)
C1—C2—C3—C6	−177.7 (4)	S2—C8—C9—S3	179.4 (2)
C2—C3—C4—C5	−1.7 (6)	S1—C8—C9—S3	1.0 (5)
C6—C3—C4—C5	178.7 (4)	C11—S4—C9—C8	178.6 (4)
C1—N1—C5—C4	0.7 (8)	C11—S4—C9—S3	−1.4 (3)
C3—C4—C5—N1	0.0 (8)	C10—S3—C9—C8	−179.1 (4)
C2—C3—C6—C7	−176.5 (4)	C10—S3—C9—S4	0.8 (3)
C4—C3—C6—C7	3.1 (7)	C9—S3—C10—C11	0.3 (4)
C2—C3—C6—S1	2.9 (6)	S3—C10—C11—S5	173.8 (2)
C4—C3—C6—S1	−177.5 (3)	S3—C10—C11—S4	−1.4 (5)
C8—S1—C6—C7	−4.2 (4)	C12—S5—C11—C10	106.0 (4)
C8—S1—C6—C3	176.3 (3)	C12—S5—C11—S4	−79.0 (3)
C3—C6—C7—S2	−179.7 (3)	C9—S4—C11—C10	1.6 (4)
S1—C6—C7—S2	0.9 (5)	C9—S4—C11—S5	−173.7 (3)
C8—S2—C7—C6	3.0 (4)	C11—S5—C12—C13	−53.5 (4)
C7—S2—C8—C9	175.7 (4)	S5—C12—C13—C14	−53.5 (5)
C7—S2—C8—S1	−5.7 (3)	C12—C13—C14—N2	152 (11)
C6—S1—C8—C9	−175.4 (4)		
